# The Study on Biocompatibility of Porous nHA/PLGA Composite Scaffolds for Tissue Engineering with Rabbit Chondrocytes *In Vitro*

**DOI:** 10.1155/2013/412745

**Published:** 2013-11-27

**Authors:** Lei Chen, Wei-Min Zhu, Zhi-Qiang Fei, Jie-Lin Chen, Jian-Yi Xiong, Ju-Feng Zhang, Li Duan, Jianghong Huang, Zhiyong Liu, Daping Wang, Yanjun Zeng

**Affiliations:** ^1^Guangzhou Medical College, Guangzhou, Guangdong 510182, China; ^2^Department of Orthopedics, Second People's Hospital of Shenzhen, Sungang West Road, Futian District, Shenzhen, Guangdong 518035, China; ^3^Shenzhen Key Laboratory of Tissue Engineering, Shenzhen, Guangdong 518035, China; ^4^Biomechanics and Medical Information Institute, Beijing University of Technology, Beijing 100022, China

## Abstract

*Objective*. To examine the biocompatibility of a novel nanohydroxyapatite/poly[lactic-co-glycolic acid] (nHA/PLGA) composite and evaluate its feasibility as a scaffold for cartilage tissue engineering. *Methods*. Chondrocytes of fetal rabbit were cultured with nHA/PLGA scaffold *in vitro* and the cell viability was assessed by MTT assay first. Cells adhering to nHA/PLGA scaffold were then observed by inverted microscope and scanning electron microscope (SEM). The cell cycle profile was analyzed by flow cytometry. *Results*. The viability of the chondrocytes on the scaffold was not affected by nHA/PLGA comparing with the control group as it was shown by MTT assay. Cells on the surface and in the pores of the scaffold increased in a time-dependent manner. Results obtained from flow cytometry showed that there was no significant difference in cell cycle profiles between the coculture group and control (*P* > 0.05). *Conclusion*. The porous nHA/PLGA composite scaffold is a biocompatible and good kind of scaffold for cartilage tissue engineering.

## 1. Introduction

Large-area cartilage defect has been difficult to treat in orthopedics for years. There are a number of ways to repair cartilage defect at present, such as cartilage graft repair, perichondrium or periosteum graft repair, and autologous or allogeneic chondrocytes graft repair. However, tissue engineering is one of the hot spots and the most promising method among others, which integrates seed cells, scaffolds, and the construction of tissue-engineered bone. Many scholars have been seeking suitable biomaterials for cartilage tissue engineering and conceived various composite materials as scaffold. Xia and his colleagues [1] investigated whether man-made porous chitosan-gelatin complex scaffold was suitable for cartilage tissue engineering in 2003. However, in most researches, cell division, proliferation, and migration have not been considered, and the effects of material degradation on cell activity have also not been investigated. Here, we introduce a new type of porous composite scaffold, nano-hydroxyapatite/poly[lactic-co-glycolic acid] (nHA/PLGA) and explore its biocompatibility with rabbit chondrocytes through *in vitro* coculture experiments.

## 2. Materials and Methods

### 2.1. Main Experimental Materials, Reagents, and Equipment

Dulbecco's Modified Eagle Medium (DMEM) (BioWhittaker, Verviers, Belgium); 20% fetal bovine serum (FBS), TRIzol Reagent, and AnnexinV-FITC/PI (Invitrogen, USA); Percoll separation solution, trypsin, penicillin, and streptomycin (Gibco, USA); MTT (3-(4,5-dimethylthiazol-2-yl)-2,5-diphenyl tetrazolium (KeyGEN, Nanjing, China)); high pressure sterilizer MLS-3780 (SANYO, Japan); clean bench VS-840 (Suzhou, China), ELIASA Model 1680 (Bio-Rad, USA); fluorescence microscopy (Leica, Germany), MIKRO 22R (Hettich, Germany); and flow cytometry (Beckman/Coulter,USA) and scanning electron microscope (TESCAN, Czech) were used in the present study.

### 2.2. Preparation of Porous nHA/PLGA Scaffolds

**Figure 1:**
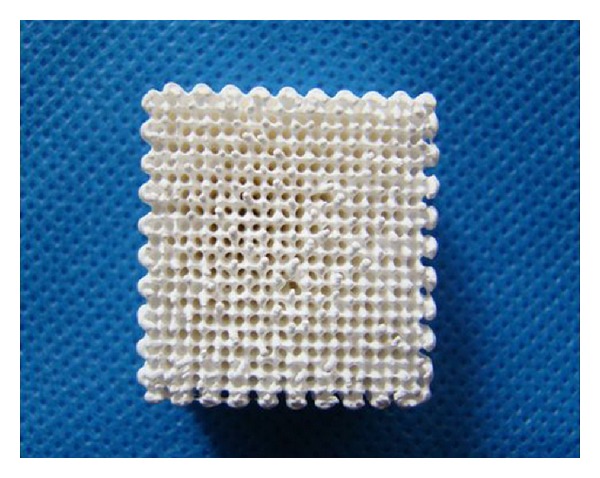
Representative of the three-dimensional porous nHA/PLGA composite sample. The scaffold is 20 mm × 20 mm × 20 mm in size. The porosity of the scaffold is 85%~90% and the pore size is between 100 and 300 microns.

Low temperature rapid prototyping technology [2] was used to prepare the three-dimensional porous PLGA/n-HA composite. One representative PLGA/n-HA composite sample was shown in Figure 1. The sample was porous and could be cut to proper size for the coculture experiment.

### 2.3. Isolation and Culture of Primary Chondrocytes from Fetal Rabbit Articular Cartilage

Three fetal rabbits born within 10 days were provided by the Medical Laboratory Animal Center of the Guangdong Province for the study. The rabbits were anesthetized to death and the cartilages were cut and rinsed with PBS plus penicillin and streptomycin (Gibco, USA) for 3 times. The surrounding connective tissues and vessels were removed and the cartilages were cut to 1 cubic millimeter. The tissues were digested with 0.25% trypsin (Gibco, USA) at 37°C for 30 min followed by the digestion with 0.2% collagenase II (Gibco, USA) for 6 h with shaking. Cells were collected after filtration and centrifugation and resuspended with DMEM/F12 (BioWhittaker, Verviers, Belgium) plus 10% FBS (Gibco, USA) and seeded at 1 × 10^5^ per mL in flasks. Media were refreshed every 3 days and cells were passaged when the confluence reached 80%. The third passage of chondrocytes was used for the co-culture experiment.

### 2.4. Coculture of Rabbit Chondrocytes and Porous nHA/PLGA Scaffold

The experiment employed control group and experimental group. For control group (group A), chondrocytes were seeded in 24-well plate alone. As for experimental group (group B), namely, coculture of cells and the scaffold, nHA/PLGA scaffolds were immersed in PBS for 5 min first and then placed in 24-well plate. Chondrocytes were then seeded at 5 × 10^5^ per well. All the plates were placed in the 37°C incubator supplied with 5% CO_2_. The media were refreshed every 3 days.

### 2.5. Cell Viability Assay

MTT assay was used to test the cell viability. Cells on day 1, day 3, day 5, day 7, and day 9 were used for the assay. 50 *μ*L MTT (KeyGEN, Nanjing, China) was added per well and incubated at 37°C for 2 h. The media were carefully removed and 200 *μ*L DMSO (Gibco, USA) was added into each well. The absorbances were measured at 490 nm using a microplate reader (BioTek, Winooski, USA).

### 2.6. Imaging and Image Analysis

A microscope (Leica, Germany) was used to observe the cells of group A and group B on day 1, day 3, and day 5. Scanning electron microscopy (SEM) was used to characterize the cell morphology on the biphasic composite. The scaffolds were fixed with glutaraldehyde, dehydrated with gradient alcohol, and dried. The scaffolds were then sputter-coated with gold and imaged using an SEM (TESCAN, Czech).

### 2.7. Flow Cytometry Analysis

Cells in both groups were collected after 7 days in culture for cell cycle analysis. Cell cycles were tested using a flow cytometer (Beckman/Coulter, USA) and the profiles were obtained by flow cytometry analysis. Proliferation index (PI) was calculated and studied by statistical analysis. PI = [(S + G2/M)/(G0/G1 + S + G2/M)] × 100%.

### 2.8. Statistical Analysis

Statistical analysis was performed by SPSS 17.0 with significance determined at *P* < 0.05. Results are reported as mean ± standard error.

## 3. Results

### 3.1. Parameters of the Scaffold

**Figure 2:**
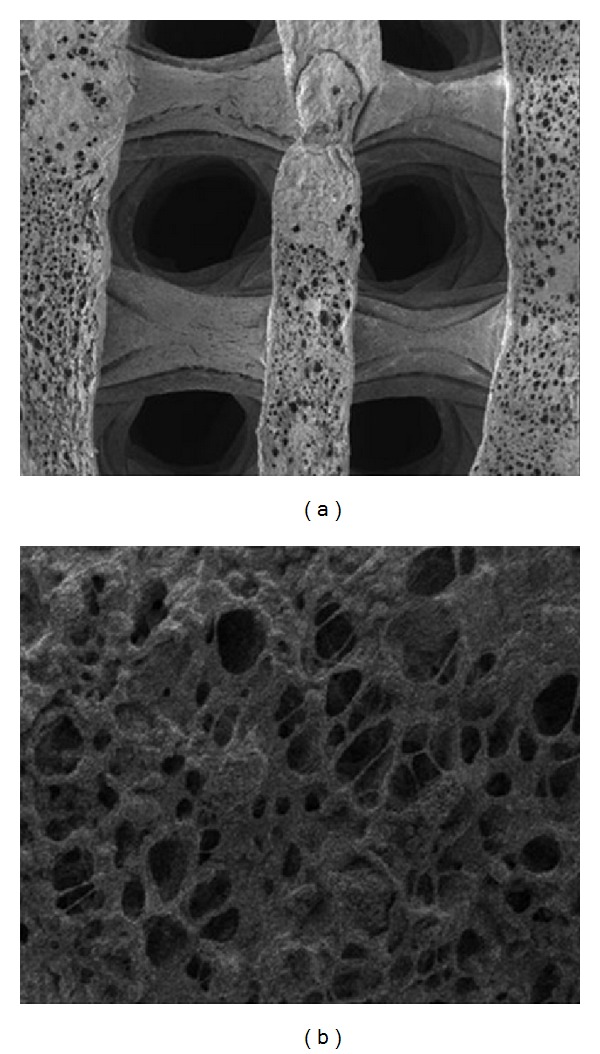
SEM images of the porous PLGA/n-HA composites. (a) SEM image of the three-dimensional porous PLGA/n-HA composite (50x). The outside diameter is around 0.6 mm. (b) SEM image of the internal structure of the PLGA/n-HA composite (1000x). The pore diameter ranges from 100 *μ*m to 300 *μ*m and the porosity reaches up to 90%.

The porous nHA/PLGA composite Scaffold is 20 mm × 20 mm × 20 mm (Figure 1) in size. The porosity was measured by alcohol soaking method and average porosity was 85%~90%. The pore size of the scaffold was measured by scanning electron microscopy (Sem), ranging from 100 to 300 microns (Figure 2(b)).

### 3.2. MTT Assay

**Figure 3:**
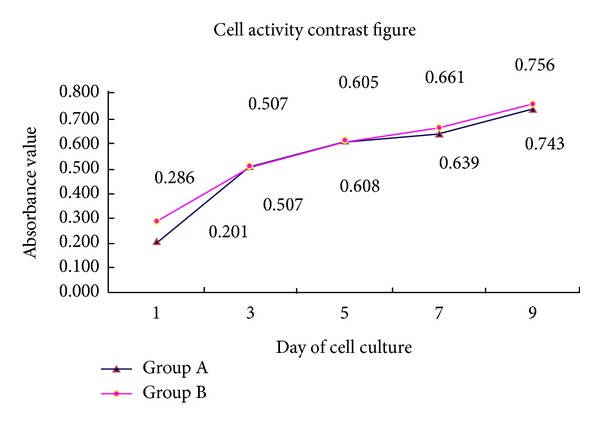
MTT assay. The absorbances were measured on day 1, day 3, day 5, day 7, and day 9. The absorbance of group A and group B increased in a similar pattern.

The cell viabilities were determined by MTT assay. As it was shown in Figure 3, no significance was observed between group A and group B (*P* > 0.05) with regard to cell proliferation capability.

### 3.3. Microscopy Observation

**Figure 4:**
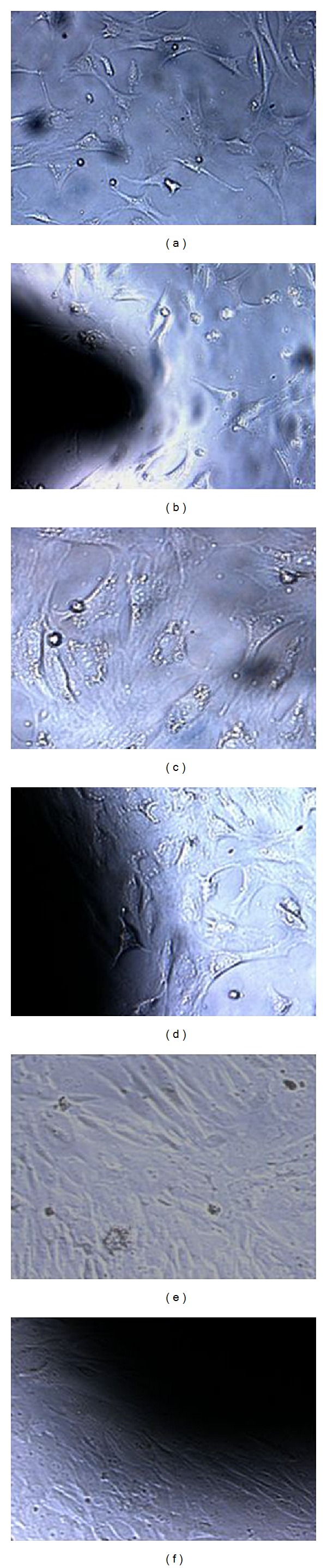
Microscopic images of cells of control group A and experimental group B at different time points (100x). After 1 day in culture, cell density in group A (a) and group B (b) was low. The cells were observed triangle-shaped, disc-shaped, and megagon-shaped. After 3 days in culture, the cells in group A (c) and group B (d) were observed to connect by cellular processes. After 5 days in culture, cells in group A (f) and group B (e) increased and completely covered the surface of the composite.

Chondrocytes in group A and group B were imaged and analyzed by inverted microscope (Figures 4(a)–4(f)). In both groups, the chondrocytes were observed triangle-shaped, disc-shaped, and megagon-shaped. The cells were connected by cellular processes. The chondrocytes observed kept proliferating and differentiating during culture.

### 3.4. Scanning Electron Microscopy (SEM)

**Figure 5:**
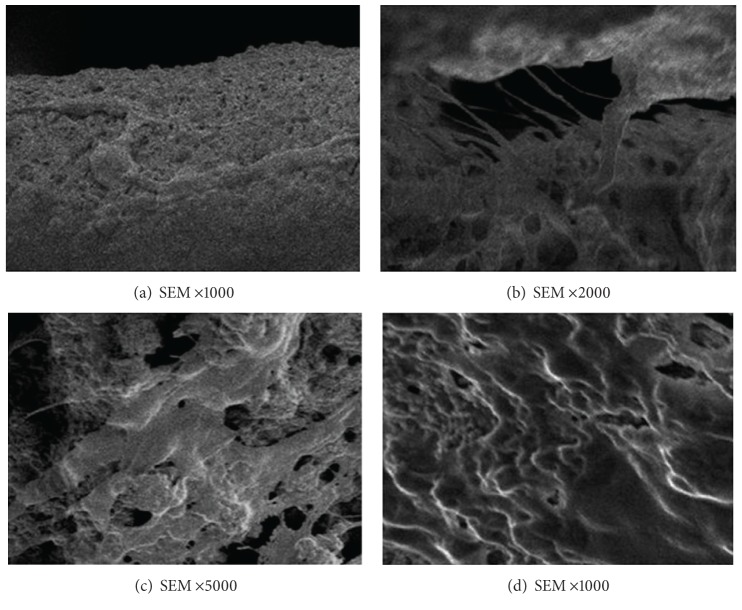
SEM images of the cocultured porous PLGA/n-HA composites with chondrocytes. (a) The chondrocytes were seeded on the composite and after 1 day in culture, cells were observed to adhere to the composite (1000x). (b) Cell processes were seen extending from the pores after 3 days in coculture (2000x). (c) Cells adhering to the composite increased after 3 days in coculture and connected with each other by processes (5000x). (d) The surfaces of the composite were fully covered by cells after 5 days in co-culture (1000x).

The images of chondrocytes grown on the scaffold were shown in Figure 5. Chondrocytes on the scaffold showed good extension capability and were fusiform-shaped. The cells increased in a time-dependent manner and gradually fused.

### 3.5. Cell Cycle Profiles

After 7 days in co-culture, chondrocytes in both groups were in normal shape and no abnormal diploid cell was observed. nHA/PLGA scaffold had little influence on cell growth. As it was shown in Table 1, no significance was observed between the two groups (*P* > 0.05).

## 4. Discussion

### 4.1. Chondrocyte

Tissue engineering has been the focus of research for long bone and cartilage defect repair in recent years. As the scaffolds are very important for tissue engineering, biocompatibility test must be carried out before use. Biocompatibility test mainly includes cell viability test, cell proliferation, and growth test on the materials [3]. Chondrocytes have been used as the seed cells for tissue engineering [4, 5]; however, dedifferentiation of chondrocytes cultured *in vitro* limited the use of passaged chondrocytes for tissue engineering [6]. Liu and his colleagues found that chondrocytes could grow well within three generations and maintain stable biological characters *in vitro* [7]. Therefore, chondrocytes of fetal rabbit limb joints within three generations were used in this experiment.

### 4.2. Preparation and Characteristics of nHA/PLGA Scaffolds

Hydroxyapatite (HA) is the most common type of bioactive materials. It is one of the inorganic compositions of human bone tissue and has good biocompatibility and bone conductibility [8]. In recent years, nanohydroxyapatite material began to be widely used in the field of tissue engineering [9], especially when it is mixed with organic compound to generate composite scaffold [10]. Polylactic-co-glycolic acid (PLGA) is currently the most used scaffold in tissue engineering, as it has good biocompatibility and controllable biodegradability. Moreover, the degraded products can be metabolized [11–13]. The nHA/PLGA scaffold material of this research is different from any other single material. It is composed of nHA and PLGA via computer-aided rapid prototyping technology and freeze-drying compound. Thus, it has the characteristics of the two kinds of materials. (1) It is more advantageous for extracellular matrix protein adhesion and interaction due to the nanostructure of material [14]. It is able to guide tissue cells to grow [15], and the biodegradability increases as the result of the increase of surface area [16]. (2) It can meet the mechanical strength of implant materials because of its higher toughness. (3) PLGA degradation products can be neutralized to some extent by nHA, thus preventing the aseptic inflammation effectively. It can also accelerate the degradation of the scaffold. (4) Its composition, shape, microstructure, and mechanical properties can be predicted in advance and it can be made to meet the clinical needs in the process of scaffold production. If porous nHA/PLGA composites with different nHA/PLGA ratios can be synthesized and different biomechanical scaffolds can be produced, not only cartilage defects but also large weight-bearing bone defect might be repaired. Li used nHA/CS composite with chondrocytes to repair rabbit articular cartilage and subchondral bone defect with good outcomes [17].

In addition, a good scaffold should have not only good biocompatibility, but also proper pore size and porosity [18]. Studies show that 200 to 350 microns are suitable for cell invasion [19] and is conductive to cell growth. For nano fiber mesh connection, it could promote infiltration of oxygen and nutrition of the body fluid inside the scaffold, which is vital to the successful repair of defects. The pore size of our nHA/PLGA porous scaffold is about 100–300 microns and the porosity is of 85%~90%, which is in full compliance with all kinds of cells.

### 4.3. Forecast

The interaction of scaffold and microenvironment cannot be explored in this experiment, as chondrocytes and scaffold were cocultured *in vitro*. *In vitro* culture system cannot completely simulate biological environment in the body, so animal experiments are needed for further *in vivo* verification. In addition, as more and more new types and suitable tissue engineering scaffolds arise for clinical applications and with the progress of the scaffold technology, tissue engineering may eventually benefit the patients.

## 5. Conclusion

The present study investigated the feasibility of nHA/PLGA being a biocompatible scaffold by cell viability test, microscope and SEM observation, and flow cytometry analysis of the cell cycle. MTT assay tested cell activity, which showed the scaffold itself had no effect on cartilage cell proliferation. Inverted microscope and SEM observation showed cell morphology at different periods of culture. With the extension of incubation time, the chondrocytes could normally adhere to the stent surface and rapidly proliferate. Flow cytometry analysis found that the scaffold had no effect on cell cycle. No abnormal diploid cell was found; thus, the scaffold materials have no tumorigenicity. In general, the nHA/PLGA porous scaffolds used in this experimental study have no adverse effects on cartilage cell adhesion, growth, proliferation, and differentiation. Therefore, the nHA/PLGA porous scaffold is with good cell compatibility and a good kind of cartilage tissue engineering scaffold.

## Figures and Tables

**Table 1:** Cell cycle analysis of coculture group and control ($\bar{x}\pm s$; *n* = 10, %).

Group	G0/G1	S	G2/M	PI
A	80.11 ± 5.71	12.04 ± 4.50	7.85 ± 2.15	19.89 ± 6.03
B	79.56 ± 5.63*	12.37 ± 4.29*	8.07 ± 2.02*	20.44 ± 5.88*

*P > 0.05, control group A, PI: proliferation index.
